# Comparative mapping in intraspecific populations uncovers a high degree of macrosynteny between A- and B-genome diploid species of peanut

**DOI:** 10.1186/1471-2164-13-608

**Published:** 2012-11-10

**Authors:** Yufang Guo, Sameer Khanal, Shunxue Tang, John E Bowers, Adam F Heesacker, Nelly Khalilian, Ervin D Nagy, Dong Zhang, Christopher A Taylor, H Thomas Stalker, Peggy Ozias-Akins, Steven J Knapp

**Affiliations:** 1 Institute of Plant Breeding, Genetics, and Genomics, 111 Riverbend Road, The University of Georgia, Athens, GA, 30602, USA; 2 Department of Horticulture, The University of Georgia, Tifton, GA, 31973, USA; 3 Department of Crop Science, North Carolina State University, Raleigh, NC, 27695, USA

**Keywords:** Peanut (*Arachis hypogaea*), SSR, Genetic linkage map, Intraspecific cross, EST

## Abstract

**Background:**

Cultivated peanut or groundnut (*Arachis hypogaea* L.) is an important oilseed crop with an allotetraploid genome (AABB, 2*n* = 4*x* = 40). Both the low level of genetic variation within the cultivated gene pool and its polyploid nature limit the utilization of molecular markers to explore genome structure and facilitate genetic improvement. Nevertheless, a wealth of genetic diversity exists in diploid *Arachis* species (2*n* = 2*x* = 20), which represent a valuable gene pool for cultivated peanut improvement. Interspecific populations have been used widely for genetic mapping in diploid species of *Arachis*. However, an intraspecific mapping strategy was essential to detect chromosomal rearrangements among species that could be obscured by mapping in interspecific populations. To develop intraspecific reference linkage maps and gain insights into karyotypic evolution within the genus, we comparatively mapped the A- and B-genome diploid species using intraspecific F_2_ populations. Exploring genome organization among diploid peanut species by comparative mapping will enhance our understanding of the cultivated tetraploid peanut genome. Moreover, new sources of molecular markers that are highly transferable between species and developed from expressed genes will be required to construct saturated genetic maps for peanut.

**Results:**

A total of 2,138 EST-SSR (expressed sequence tag-simple sequence repeat) markers were developed by mining a tetraploid peanut EST assembly including 101,132 unigenes (37,916 contigs and 63,216 singletons) derived from 70,771 long-read (Sanger) and 270,957 short-read (454) sequences. A set of 97 SSR markers were also developed by mining 9,517 genomic survey sequences of *Arachis*. An SSR-based intraspecific linkage map was constructed using an F_2_ population derived from a cross between K 9484 (PI 298639) and GKBSPSc 30081 (PI 468327) in the B-genome species *A*. *batizocoi*. A high degree of macrosynteny was observed when comparing the homoeologous linkage groups between A (*A*. *duranensis*) and B (*A*. *batizocoi*) genomes. Comparison of the A- and B-genome genetic linkage maps also showed a total of five inversions and one major reciprocal translocation between two pairs of chromosomes under our current mapping resolution.

**Conclusions:**

Our findings will contribute to understanding tetraploid peanut genome origin and evolution and eventually promote its genetic improvement. The newly developed EST-SSR markers will enrich current molecular marker resources in peanut.

## Background

Peanut or groundnut (*Arachis hypogaea* L.) is both an important oilseed crop and a direct protein source for human nutrition and is the only domesticated species in the genus *Arachis*. It is an allotetraploid (2*n* = 4*x* = 40, AABB) with one pair of distinctively small chromosomes [1], and was believed to have originated from a single hybridization event at least 3,500 years ago [2-4]. This genus also contains additional tetraploid, diploid (2*n* = 2*x* = 20) and aneuploid (2*n* = 2*x* = 18) species. *Arachis hypogaea* is a member of section *Arachis*, which contains species with A, B, or D genomes. The *Arachis* genome is characterized by the presence of a small pair of chromosomes with a lower level of euchromatin condensation [5], whereas the B genome is distinguished by the presence of a pair of chromosomes with a secondary constriction proximal to the centromere [6], and has recently been divided into subgroups [7]. *Arachis duranensis* and *A*. *ipaënsis* are most likely the ancestral A- and B-genome species of cultivated peanut, respectively [2,5,8-10]. The D genome is represented by the single species *A*. *glandulifera*[11,12].

Due to both the low level of genetic variation within the cultivated gene pool and the polyploid nature of cultivated peanut, relatively fewer numbers of genetic linkage maps have been constructed as compared to many other crop species [13]. Considering its relatively large genome size (2800Mb/1C), there is still great need to improve marker density and genome coverage [14,15]. For example, mapping of quantitative trait loci (QTL) for late leaf spot, rust [16] and seed quality [17] traits has only been possible at low marker density with 225 and 45 SSR loci, respectively.

A wealth of genetic diversity exists in diploid *Arachis* species [18] with the potential to introgress novel alleles into cultivated peanut [4,19]. The diploid wild species are not commercially important in the food industry, but they provide a valuable gene pool for sources of resistance to many economically important pathogens and pests [20-23]. Several linkage maps have been constructed in both A- and B-genome diploid species [24-28], with the first A-genome linkage map reported being composed of 11 linkage groups with 117 RFLP loci and with a genome coverage of 1,063 cM [26]. Subsequent A-genome linkage maps consisted of 167 RAPD and 39 RFLPs, 170 SSR, or 369 assorted markers [25,27,28]. An interspecific B-genome linkage map has been constructed with 149 SSR loci covering 1,294 cM [24]. Synteny between A and B genomes was compared using diploid as well as synthetic amphidiploid linkage maps [24,29,30].

The above-referenced diploid linkage maps were generated from interspecific crosses. Thus, the detection of chromosomal rearrangements within species could be obscured, and comparative A- and B-genome linkage maps could be complicated due to chromosomal rearrangements associated with speciation [31-35]. Previous comparisons were based on limited numbers of orthologous loci and markers that were primarily derived from genomic sequences. Therefore, map coverage and resolution need to be improved using more function-related and highly transferable markers such as EST-SSRs that facilitate comparative and evolutionary genomics studies. To enrich the currently available SSR marker resources in peanut and gain clearer insights into karyotypic evolution within the genus, a new set of EST-SSR markers was developed and mapped in an intraspecific B-genome mapping population. Comparative mapping to intraspecific A-genome linkage maps revealed a high degree of macrosynteny.

## Results and discussion

### Marker development

A total of 101,132 unigenes representing ca. 37 Mb of the *A. hypogaea* genome (Additional file 1) were mined for SSRs. We found 7,413 perfectly repeated di-, tri-, tetra-, penta-, and hexa-nucleotide motifs (7.3% of the unigenes contained SSRs). The SSR frequency in the above EST resources is comparable with previous reports in cultivated peanut [36,37], and wild *Arachis* species [38]. The overall SSR density was 3,190 bp per Mb and corresponded to approximately 1 per 5 kb of the genic region, which is similar to a previous report of 1/5.5 kb in cultivated peanut [15]. higher than *Arabidopsis*[39], and barley [40] but slightly lower than that reported for rice [41] and pepper [42], yet within the range of most other plant species (~5%) [43]. The average SSR length was about 16 bp with almost 88% of SSRs shorter than 22 bp. Among repeat motifs, dinucleotides were predominant (53.3%), which was inconsistent with the study of Koilkonda et al. [15], who found trinucleotide repeats to be the most abundant (66.8%). Discrepancies observed in various studies could be explained by the degree of representation of dinucleotide rich UTRs in the genic sequences used or by EST database mining software and SSR search criteria [43]. Dinucleotide repeat motifs were predominantly distributed in the UTRs while trinucleotide repeat motifs were more frequent in exons. Considering the mode of slippage-mediated mutations, it is unlikely that a large proportion of the dinucleotides would be present in the coding regions; mutations in trinucleotides or their multiples would only be tolerated if they do not disturb the open reading frame.

In our study, the most common dinucleotide repeat motif class was (AG)n (61.0%), while the least common repeat class was (CG)n (1.2%). Similarly, the most abundant trinucleotide motif class was (AAG)n (35.2%), and the least frequent was (CGA)n (0.9%). Motif classes (CG)n and (CGA)n are also relatively infrequent in other plants as well as animals [44]. The motif types (AG)n and (AAG)n have been reported as the most common di- and tri-nucleotide repeats identified in other plant EST databases [43,45-47], including peanut (*A*. *hypogaea*) [15,30,37,40,48,49].

We designed 2,138 EST-SSR primer pairs from the identified SSRs (Additional file 2), with 94.6% of them targeted to amplify perfect repeats and over 70% of the primers targeted at trinucleotide motif types. By screening a panel containing eight genotypes, (Additional file 3), 15.3% of the 2,138 primer pairs didn't amplify any interpretable fragments, and 82.7% of them were fully transferable between tetraploid and diploid species. As expected, we observed a relatively higher transferability for EST-SSRs than for genomic SSRs; this is most likely due to greater sequence conservation within expressed regions among related species compared to non-coding regions [25,36,43,50,51]. The frequency of polymorphism among the four tetraploid genotypes was 11.2% and less than 10.0% between paired tetraploids. The polymorphism between two *A. duranensis* accessions was 41.9% while between two *A. batizocoi* accessions was 21.3% (Additional file 4). The polymorphisms for our intraspecific diploid mapping population parents were comparable to the previous reports of interspecific diploid mapping populations for EST-SSR markers [24,25,30]. We also observed that the polymorphism was higher in the A-genome species than in the B-genome species [52].

Our findings also support the general theory that the degree of polymorphism of the SSR marker increases with the total length of the repeat [25,53,54]. A positive correlation was observed between repeat length and polymorphism rate, but the trend seemed more obvious in tetraploid than in diploid genotypes (Additional file 5), which was supported by the previous observations of Moretzsohn et al. [25]. For example, SSRs with a repeat length >26 bp showed up to 30% polymorphism between the four tetraploid genotypes, while less than 15% polymorphism was observed for SSRs with repeat length <20 bp. However, when all eight genotypes were combined, no trends were observed between the polymorphism and repeat length. For tetraploid genotypes, AG/CT repeats were more polymorphic than GT/CA, while in diploid genotypes, this effect can only be observed between interspecific genotypes in spite of the fact that the polymorphisms among four diploid genotypes were overall high (around 70%) (Table 1). Similar observations that AG/CT repeats were more polymorphic than GT/CA were previously reported in peanut [25,36,48]. The polymorphisms for dinucleotide repeat motif types were generally higher than for trinucleotide repeat motif types. However, no consistent pattern emerged for ranking of polymorphism rate by motif type.

**Table 1:** Effect of SSR repeat motif types on frequency of polymorphism among tetraploid and diploid genotypes

**Motif type**	**No. of markers**	**Between Tif-runner and GTC20 (%)**	**Between NC94022 and SunOleic (%)**	**Among four tetraploid (%)**	**Between 30081 and 9484 (%)**	**Between Grif 15036 and PI 475887 (%)**	**Among four diploid (%)**	**Among eight genotypes (%)**
GT/CA	285	8.1	2.7	10.8	21.6	51.4	75.7	78.4
AG/CT	37	16.8	14.0	24.2	32.3	57.5	70.2	73.0
AT	69	14.5	13.0	23.2	21.7	47.8	69.6	76.8
CG	1	0	0	0	0	0	0	0
CCG/CGG	41	2.4	2.4	4.9	7.3	29.3	56.1	61.0
ACC/GGT	149	3.4	3.4	5.4	19.5	39.0	67.8	73.2
ACG/CGT	55	3.6	0	3.6	14.5	27.3	60.0	67.3
AGC/GCT	52	0	1.9	3.8	11.5	25.0	59.6	65.4
AGG/CCT	107	3.7	1.9	6.5	10.3	26.2	55.1	65.4
AAC/GTT	141	5.7	5.0	9.2	17.0	43.3	70.2	78.7
AAG/CTT	519	5.4	4.8	9.6	24.5	39.1	66.5	74.2
ACT/AGT	130	2.3	3.8	6.2	12.3	34.6	63.8	73.1
ATC/GAT	127	5.5	5.5	7.9	13.4	39.4	67.7	75.6
AAT/ATT	168	6.0	3.6	6.0	20.2	44.6	70.8	79.2

In addition to ESTs, a total of 9,517 genome survey sequences (GSSs) representing ca. 5.5 Mb of the *Arachis* genome were mined for SSRs. Overall, 1,168 perfectly repeated di-, tri-, or tetra-nucleotide motifs were identified from 960 unique sequences. By SSCP screening, 97 SSR primer pairs can produce reliable amplification across a panel of 12 genotypes representing different species (Additional file 6).

### Genetic mapping

Collectively, 2,138 newly developed EST-SSR primer pairs (Additional file 2), 97 genomic SSR markers developed from genome survey sequences and 612 genomic SSR primer pairs in the public domain (Additional file 7) were screened for polymorphisms between the parents of an intraspecific *A*. *batizocoi* (BB, 2*n* = 2*x* = 20) F_2_ mapping population. Although *A*. *ipaënsi*s is the more likely B-genome donor than *A*. *batizocoi* to tetraploid peanut species *A. hypogaea*[52,55,56], *A*. *batizocoi* retains a high level of similarity to the B subgenome of cultivated peanut [57]. For example, the F_1_ plant derived from crossing *A*. *hypogaea* by a synthetic amphidiploid (*A*. *batizocoi* × *A*. *duranensis*) produced bivalents, and a few F_4_ plants from this cross were even able to produce two-seeded pods [58]. Furthermore, a diversity study indicated that among all the B-genome species, *A*. *batizocoi* showed the second closest relationship to *A*. *hypogaea*, after *A*. *ipaënsi*s [55]. Although the hypothetical B-genome donor *A*. *ipaënsi*s was not used for linkage mapping in this report because only a single accession is available in the U.S. germplasm collection, our *A*. *batizocoi* intraspecific map should still provide a very close representation of the B-genome donor of tetraploid peanut.

The screening of *A*. *batizocoi* accessions 9484 and 30081 produced 455 polymorphic EST-SSR and 171 polymorphic genomic SSR markers. After excluding makers with numerous and/or faint bands and abnormal segregation ratios (markers that showed extreme segregation ratios were assumed to be caused by loci with indistinguishable bands), a total of 481 markers were used for linkage map construction. Of these, 449 loci (including 347 loci from the newly developed EST-SSR markers, 14 loci from genomic SSR markers developed from GSS sequences, and 88 loci from the genomic SSR markers already reported [18,25,48,49,59-62]) were mapped into 16 linkage groups (LGs), 14 of which aligned with the 10 chromosome pairs of diploid peanut numbered according to colinearity with *A*. *duranensis* (A- genome) linkage groups from Nagy et al. [63]. The remaining two small linkage groups had no common markers with A- genome linkage groups, thus their chromosomal locations are unknown. Their lengths were 1.1 cM and 9.2 cM, respectively. One LG consisted of markers GM2227 and GM1611, and another LG was composed of markers GM1241 and GM748.

**Figure 1:**
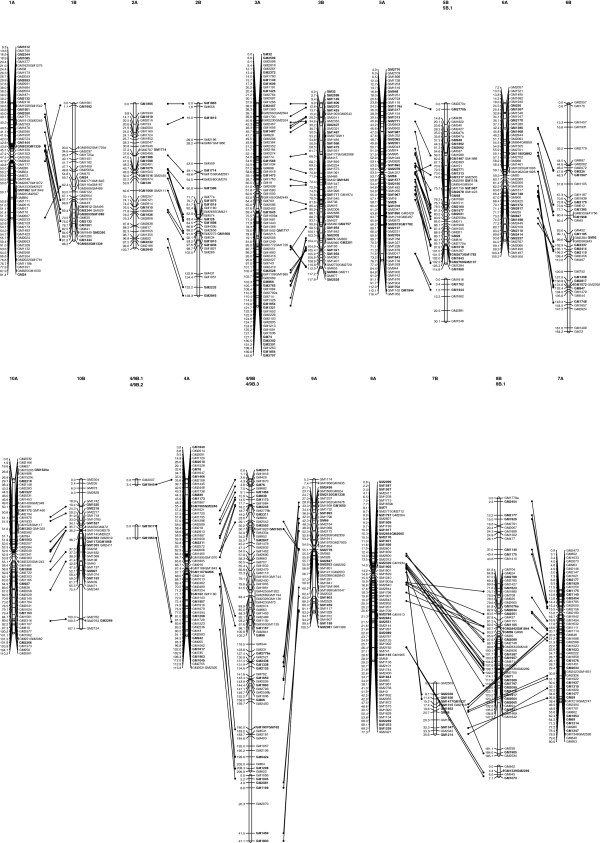
**The alignment of A- and B- genome linkage maps of *Arachis.*** Alignment of B- genome linkage groups with A- genome linkage groups, orthologous markers are highlighted in bold. The B- genome map was based on EST-SSR markers and genomic SSR markers, and was obtained through the analysis of 94 F_2_ plants from the intraspecific crossing of *A*. *batizocoi*. The A- genome map was based on EST-SSR markers and genomic SSR markers, obtained through the analysis of 94 F_2_ plants from the intraspecific crossing of *A*. *duranensis*. The dotted lines indicate the correspondences between loci.

Overall, the linkage map covered 1,278.6 cM, with marker densities ranging from 1.1 cM/locus in LG11 to 9.2 cM/locus in LG12, giving an average density of 2.9 cM/locus for the entire map (Table 2, Figure 1). The LGs ranged from 1.1 to 210 cM in length, and had two (4/9B.1, 4/9B.2, and the two linkage groups described above) to 80 (4/9B) marker loci. Gaps larger than 30.0 cM were observed only at the end of 1B (31.9 cM) and 8B (33.6 cM) (Figure 1). The number of linkage groups observed in this study is larger than the number of haploid chromosomes in the diploid species (n = 10), which may be due to insufficient markers for the chromosome coverage. Furthermore, since the B-genome linkage groups were named by comparing common markers to A-genome linkage groups, the chromosome location of some small linkage groups could not be identified if they lacked anchor markers. The map length was comparable to previously published diploid peanut genetic maps while the density was the highest among all B-genome linkage maps constructed to date [24-26]. This is by far the most saturated map constructed in a B-genome diploid peanut, and also represents the first intraspecific map of a B-genome species.

**Table 2:** Number of loci, map length and density of each linkage group in the 9484x30081 map

**Linkage group**	**Length (cM)**	**No. of marker/LG**	**Density (cM/locus)**	**Number of distorted loci (α = 0.05)**
1B	98.2	38	2.7	10
2B	138.3	35	4.1	1
3B	117.8	62	1.9	0
4/9B	209.9	80	2.7	1
4/9B.1	3.4	2	3.4	0
4/9B.2	8.6	2	8.6	0
4/9B.3	47.1	7	7.9	1
5B	114.8	52	2.3	0
5B.1	30.1	6	6.0	0
6B	164.2	44	3.8	1
7B	36.6	13	3.1	2
8B	185.1	60	3.1	0
8B.1	7.1	5	1.8	0
10B	107.1	39	2.8	2
LG11	1.1	2	1.1	0
LG12	9.2	2	9.2	0
Whole map	1278.6	449	2.9	18 (4.0%)

Significant segregation distortion (α ≤ 0.05) was observed for 18 (4.0%) marker loci, distributed in seven linkage groups representing six B-genome chromosomes (Additional file 8), which was much lower than previously reported segregation distortion (21.5%) in an interspecific B-genome linkage map [24]. Similarly, high levels of skewed segregation in interspecific crosses compared to intraspecific crosses have been reported in both barley and cowpea [64,65]. In the *A. batizocoi* map, a total of 11 markers skewed towards parental line 9484 and two towards 30081. Linkage group 1B contained the largest number of distorted marker loci (10 marker loci comprising 55.6% of the total distorted markers). All the marker loci on this linkage group skewed towards 9484 and clustered within a 37.1 cM genomic region, which is 37.8% of the total mapping distance for 1B and 26.3% of the marker loci for this chromosome. Linkage groups 3B, 5B, and 8B had no markers showing segregation distortion (Additional file 8).

### Synteny between A-genome and B-genome linkage maps

The B-genome linkage groups were aligned to A-genome linkage groups by 167 putative orthologous marker loci, 10 to 25 in each chromosome (Table 3). Compared to previous reports, which identified 51 shared markers between the A- and B-genome maps [24], or 53 SSR markers that mapped on both A and B subgenomes in the tetraploid map [30], our comparisons are based on a higher density, higher information content map, and should more precisely position chromosomal rearrangement events within defined genomic regions. Syntenic segments were supported by multiple markers in the same linear order with some incongruities reflecting simple translocations and inversions. A high degree of macrosynteny was observed when comparing the nine major linkage groups identified in the B genome to the 10 major linkage groups (1A-10A) in the A genome (Figure 1). Linkage groups 5B and 5B.1 can both be aligned with linkage group 5A, with putative orthologous markers showing colinearity. Similarly, LGs 8B and 8B.1 can both be aligned with linkage group 8A. Since the subgroups couldn’t be merged into one linkage group with a minimum LOD threshold of 3.0 without a >50 cM gap between the adjacent subgroup loci, the two subgroups were displayed separately.

**Table 3:** Number of common markers between the corresponding linkage groups from A- and B-genome genetic maps

		Linkage group	1A	2A	3A	4A	5A	6A	7A	8A	9A	10A	Total
		No. of marker loci	73	40	89	67	66	51	56	75	55	61	633
Linkage group	No. of marker loci												
1B	38		12										
2B	35			13									
3B	62				22								
4/9B*	91					18					13		
5B**	58						25						
6B	44							15					
7B	13								7	2			
8B***	65								9	21			
10B	39											10	
Total	445												167

*4/9B including 4/9B, 4/9B.1, 4/9B.2, and 4/9B.3.

**5B including 5B and 5B.1.

***8B including 8B and 8B.1.

#### Completely syntenic chromosomes

For LGs 2A and 2B, all 13 putative orthologous markers were colinear, covering a map distance of 138.3 cM (100%) on B2 and 103.7 cM (100%) on A2. Therefore, 2B and 2A showed a high degree of macrosynteny as previously reported [24], although we did not detect any split correspondence relationship with A2 and B10 as in the previous report [24]. Ten putative orthologous markers between 10A and 10B indicated well defined macrosynteny. The common markers spanned 81.1 cM on 10B and 75.0 cM on 10A, accounting for 75.7% and 68.0% of the total linkage group length, respectively.

#### Chromosomes with inversions

The 12 putative orthologous markers between 1A and 1B were clustered into two chromosome segments. One segment was colinear, containing 10 putative orthologous markers with 28.2 cM map coverage on 1B and 34.5 cM on 1A, accounting for 28.7% and 37.7% of the total lengths, respectively. The other chromosome fragment had a reversed colinear order, with two putative orthologous markers spanning 58.5 cM on 1B and 40.7 cM on 1A, accounting for 59.6% and 44.5% of the total lengths, respectively. The reversed region defined by two putative orthologous markers on both 1A and 1B indicated a likely inversion between these two chromosomes. This observation was similar to previous reports [24,30].

For LGs 3B and 3A, 22 common markers were clustered into two chromosomal segments. The first of these was extensively colinear between 3A and 3B, with 12 putative orthologous markers spanning 88.7 cM (75.3%) on 3B and 74.9 cM (51.4%) on 3A. The other fragment had a reversed order with 10 putative orthologous markers spanning 20.9 cM (17.7%) on 3B and 43.2 cM (29.7%) on 3A. The chromosome segment inversion between 3A and 3B has not been previously reported.

For LGs 5A and 5B, there were 25 putative orthologous markers in total, spanning the entire 5B, 4.1 cM (13.6%) of 5B.1, and 108.0 cM (92.8%) of 5A, with a generally colinear order, except for a chromosome segment with nine putative orthologous marker loci spanning 31.4 cM (27.4%) in 5B, and 49.0 cM (42.1%) in the reversed order for 5A. Since the inverted chromosome segments accounted for nearly 30% of the total linkage groups’ lengths and 36.0% (9 out of 25) of the total putative orthologous markers in both A and B genomes, there could be an inverted chromosome segment between 5A and 5B, which also has not been detected from previous reports.

Between chromosomes 6A and 6B, there were 15 putative orthologous markers that spanned 95.6 cM in 6B and 69.1 cM in 6A, accounting for 58.2% and 65.6% of the total lengths on the linkage groups, respectively. Seven putative orthologous markers were mapped in a colinear manner at the top part of both linkage groups. Segments containing eight putative orthologous markers at the bottom part were involved in an inversion event. The inverted chromosome segment on 6B was 40.5 cM long and accounted for 24.7% of the entire 6B. The corresponding segment on 6A was 23.2 cM, accounting for 22.0% of the entire 6A. This inversion was reported previously [24], but they also reported the split correspondence relationships between B6 and A10, which was not revealed in our study.

#### Complex chromosome rearrangements

In addition to the observed simple inversion events, we also found more complex chromosome rearrangements. This intraspecific *A*. *batizocoi* genetic map has nine major linkage groups instead of the expected 10. When the A- and B-genome linkage groups were aligned, one of the major B-genome linkage groups was found to correspond to both 4A and 9A and was therefore named as 4/9B. Another smaller linkage group also had putative orthologous markers with 4A and 9A therefore it was named 4/9B.3. The linkage group 4/9B contains 80 markers spanning 209.9 cM, while 4/9B.3 contains 7 markers spanning 47.1 cM. In addition, there were two smaller LGs, 4/9B.1 and 4/9B.2, containing two markers each with genetic distances of 3.4 cM and 8.6 cM, respectively. They were also designated as fragments of 4/9B according to their putative orthologous markers with 4A and/or 9A.

There were several reasons that we did not further separate 4/9B into 4B and 9B. Firstly, these linkage groups remained inseparable even when increasing the LOD threshold to 20. Secondly, the markers were located densely and evenly, with no obvious gaps between two chromosome fragments. The average marker density was 2.7 cM/locus on 4/9B and 7.9 cM on 4/9B.3, which was comparable with the rest of the genome. Thirdly, the effects on map order due to distorted segregation were minimal. There was only one marker that was distorted on each of 4/9B and 4/9B.3. Double crossover events were evaluated alongside 4/9B and 4/9B.3 but no unusual segregating markers or marker blocks were observed. Fourthly, when aligning 4/9B, 4/9B.1, 4/9B.2 and 4/9B.3 with 4A and 9A separately, by using only putative orthologous markers, 4A with 4/9B, 4/9B.1, 4/9B.2 and 4/9B.3 showed complete colinearity, while 9A and 4/9B, 4/9B.1, 4/9B.2 and 4/9B.3 showed a possible inversion. Synteny between 4A and 4B had been reported by Moretzsohn et al. [24] and Fonceka et al. [30]. An inversion between 9A and 9B was also consistent with a previous report [30]. Lastly, all putative orthologous markers between 4A and 9A were interspersed alongside 4/9B, 4/9B.1, 4/9B.2 and 4/9B.3. From a previous cytological study of the intraspecific variability of *A*. *batizocoi* using five accessions [66], hybrids between accessions 30081 and 9484 had reduced pollen stainability (88.6%). Moreover, cytogenetic analyses of F_1_s from the same cross showed a low frequency of quadrivalents [11,66], indicating a reciprocal translocation that would cause pairing between two non-homologous chromosomes during meiosis. The similarity between the two chromosomes involved in reciprocal translocation could explain the integrated linkage group 4/9B in our study.

A quadrivalent relationship was observed when aligning 7B and 8B with 7A and 8A. On linkage group 7B, there were two colinearly located putative orthologous markers with 7A, covering 3.3 cM and accounting for 9.0% of the total length on 7B. The bottom segment had seven colinearly located putative orthologous markers with 8A, spanning 21.4 cM, about 58.5% of the entire 7B. On 8B, a colinear fragment with 7A was located from 2.2 cM (from the top) to 91.2 cM (from the top), with nine putative orthologous markers spanning a genetic distance of 89.0 cM (48.1% of 8B), whereas the other 18 putative orthologous markers with 8A were colinearly located from 67.2 cM to 184.0 cM (also from the top of the linkage group), spanning a genetic distance of 116.8 cM (63.1% of 8B). Moreover, three colinear putative orthologous markers with 8A also were found on 8B.1. This may indicate a reciprocal translocation between 7B and 8B. Similar translocations on corresponding linkage groups were found both at the diploid level [24] and tetraploid level (from the crosses of a tetraploid variety with a tetraploid AABB amphidiploid) [30]. However, the previous reports did not identify the correspondence between LGs a07 (corresponding to LG 7A in our study) and b08 (corresponding to LG 8B in our study), which might due to their relatively shorter chromosome coverage on LG 8B (29.8 cM in the synthetic amphidiploid map and 86.4 cM in the diploid map versus 192.2 cM in this report) [24,30]. Therefore, the rearrangement is most likely a reciprocal translocation, and may have contributed to the divergence of A and B genomes, perhaps as an ancient event that occurred before peanut polyploidization and remained subsequent to tetraploidization of cultivated peanut [30]. Although the chromosomes of *A*. *hypogaea* have differentiated botanical varieties and individual lines that can be separated based on location of the secondary constriction and symmetry of chromosomes [67], introgression of disease and insect resistance traits from wild species into the cultivated peanut has been successful [68].

#### Comparative mapping summary

Comparison of the genetic linkage maps of A and B genomes indicated that the chromosomal differences between these two species could be explained by a total of five inversions and one reciprocal translocation under our current mapping resolution. Because of the possible minor differences in ordering of tightly linked markers, confident identification of small inversions is more difficult than translocations. To detect the confidence of inversion, we compared our predicted inversions with previously published maps. Moretzsohn et al. [24], used different A- and B-genome diploid species and showed four inversions and one translocation by comparing diploid A- and B-genome maps from interspecific mapping populations. Fonceka et al. [30] used a synthetic allotetraploid population to compare linkage maps of the A and B subgenomes, which revealed at least three inversions, while Burow et al. [29] revealed four inversions. Our study identified additional translocation events by using intraspecific diploid mapping populations, and also detected a reciprocal translocation within the B-genome species *A*. *batizocoi*.

Chromosomal rearrangements are common within and among A- and B-genome diploid species. For example, in an analysis to determine the intraspecific variability within the B-genome species *A*. *batizocoi*[66], quadrivalents, hexavalents and octavalents were observed during meiosis in F_1_ hybrids of different accessions, indicating one to three reciprocal translocations that differentiate these accessions. Thus, karyotypic evolution via translocations was considered to be an important mechanism for species differentiation [66]. In our study, the linkage map from *A*. *duranensis* was used as the reference map from which the chromosome rearrangements between A and B genomes were inferred [63]. However, chromosomal rearrangements within the A-genome could exist when considering the higher genetic diversity among the various accessions of the A-genome species, *A*. *duranensis*. Previous research found a low frequency (0.01-0.26/PMC) of multivalents in 12 of 27 hybrids from crosses of *A*. *duranensis* accessions [69], and quadrivalents were identified in all these 12 hybrids, which likely represents chromosome translocations within this species. The asymmetrical chromosomes found in different accessions, furthermore, indicated the presence of translocations. In addition, varied fertility of F_1_s (from less than 4.7% to greater than 95%) and the diverse morphological traits also indicated wide genetic diversity in *A*. *duranensis*[12,69]. Univalents, laggards, and multivalents can all be detected in intraspecific A-genome and interspecific A- by B-genome F_1_ hybrids, indicating the prevalence of chromosomal rearrangements in peanut diploid species. We herein verify by genetic mapping that a chromosomal translocation has occurred within the B-genome species *A*. *batizocoi*. Our findings of one reciprocal translocation between chromosomes 7 and 8 in A-genome species *A*. *duranensis* and B-genome species *A*. *batizocoi* is consistent with previous reports [24,30]. The slight discrepancy of the number of inversions between A- and B-genome chromosomes could be due to the genetic variation of the different accessions/species used in the mapping population development.

## Conclusions

The present study developed and characterized an extensive set of EST-SSR and genomic SSR markers. Comparative mapping of our intraspecific A- and B-genome populations showed a high degree of macrosynteny between A- and B-genome diploid species of peanut. Consistent with previous cytological studies, it was evident that chromosomal rearrangements were common within and between both A- and B-genome diploid species. Karyotypic evolution via translocations could be an important mechanism for differentiation of the species. Our findings will facilitate an understanding of tetraploid peanut genome origin and eventually promote its genetic improvement.

## Methods

### Plant materials

#### Mapping population

An F_2_ population consisting of 94 plants was developed by selfing four F_1_ plants from the intraspecific cross of two *A*. *batizocoi* lines PI 298639 (accession no. K 9484) and PI 468327 (accession no. GKBSPSc 30081). The A-genome F_2_ mapping population also consisted of 94 plants from the intraspecific cross of *A. duranensis* PI 475887 and Grif 15036 [63].

#### Plant materials for EST-SSR characterization

Eight genotypes including four tetraploid and four diploid accessions were used to screen all 2,138 markers. The four tetraploid genotypes in the screening panel included Tifrunner (*A. hypogaea* subsp. *hypogaea* var. *hypogaea*), a runner-type peanut cultivar; GT-C20 (*A. hypogaea* subsp. *fastigiata* var. *vulgaris*), a Spanish exotic accession with reduced aflatoxin contamination; NC94022 (*A. hypogaea* subsp. *hypogaea*), an exotic accession with higher resistance to TSWV (tomato spotted-wilt virus) derived from var. *hirsuta*; and SunOleic 97R (*A. hypogaea* subsp. *hypogaea* var. *hypogaea*), a high oleic runner-type cultivar. For the diploid genotypes, PI 475887 and Grif 15036 are A-genome germplasm accessions of *A. duranensis*, while accessions 9484 and 30081 are B-genome germplasm accessions in *A. batizocoi*. They are parental lines of the respective A- and B-genome mapping populations.

### SSR discovery, marker development, and length polymorphism screening

#### Mining the peanut EST database for SSRs

The sequence database used for SSR marker development harbors a total of 70,771 long-read (Sanger) ESTs and 270,957 short-read (454) ESTs assembled into 101,132 unigenes (Accession: PRJNA49471; Additional file 1). Unigenes in the transcript assembly were screened for perfect repeat motifs using SSR-IT ( http://www.gramene.org/db/markers/ssrtool) and for imperfect motifs using FastPCR ( http://primerdigital.com/fastpcr.html). The repeat count (n) threshold for each motif type was set for n ≥ 5. Information on repeat motif, repeat number, and SSR start and end positions within the respective ESTs were extracted from the SSR-IT output. The grouping of SSR motifs into respective repeat classes was performed following the method of Jurka and Pethiyagoda [70]. Flanking forward and reverse primers were designed using Primer3 ( http://frodo.wi.mit.edu) [71]. The parameters were set as follows: primer length from 19 to 23 with 21 nucleotides as optimum; amplification size of 100 to 400 base pairs; annealing temperatures from 59°C to 63°C with a maximum difference of 3°C; and GC contents from 25% to 45%.

#### Mining genome survey sequences (GSSs) for SSRs

Methylation filtered (ME) and unfiltered (U) genome libraries were constructed from *A*. *duranensis*, *A*. *batizocoi*, and *A. hypogaea* by Orion Genomics (Saint Louis, Missouri) [72]. A total of 9,517 unique genome survey sequences (GSS) were used for mining SSRs.

Similarly, SSR-IT was used to screen for perfect repeat motifs ( http://www.gramene.org/db/markers/ssrtool[47]), and Primer3 ( http://frodo.wi.mit.edu) [71] was used for primer design. Primers were screened for overall amplification quality against 12 genotypes, including four diploid and eight tetraploid accessions (Additional file 6), and with SSCP gels by silver staining according to protocols described previously [73].

### SSR marker genotyping

Genomic DNA was isolated from young leaves by a modified cetyltrimethylammonium bromide (CTAB) method [74]. SSR markers were genotyped on an ABI3730XL Capillary DNA Sequencer (Applied Biosystems, Foster City, CA) using forward primers labeled with FAM, HEX, or TAMRA fluorophores. PCR was performed in a 12 μL reaction mixture containing 1.0× PCR buffer, 2.5 mM Mg^++^, 0.2 mM each of dNTPs, 5.0 pmol of each primer, 0.5 unit of *Taq* polymerase, and 10 ng of genomic DNA. Touchdown PCR was used to reduce spurious amplification [75]. The SSR markers were screened for amplification and length polymorphisms using GeneMapper 3.0 software (Applied Biosystems, Foster City, CA).

### Genetic mapping, macrosynteny analysis, and cMap database construction

A total of 481 polymorphic markers were used to screen 94 F_2_ progenies for map construction. Segregation distortion at each marker locus was tested against the expected segregation ratios (1:2:1 for codominant markers and 3:1 for dominant markers) using a chi-square goodness of fit test. Genetic maps were constructed using Mapmaker 3.0, with error detection on [76,77]. The initial linkage groups were first determined using the “group” application with a minimum likelihood odds (LOD) threshold of 15 and a maximum recombination fraction (θ) of 0.35. After aligning the draft map with the A-genome map [63], the LOD score was relaxed to 5 with θ =0.35 for a second analysis to merge the linkage groups that could align with the homoeologous A-genome linkage groups. Map distances (cM) were calculated using the Kosambi mapping function [78]. The “try”, “compare”, and “ripple” commands were used to confirm the marker order. Mapchart 2.2 was used for the graphic visualization of the linkage groups [79].

The *A*. *batizocoi* (B-genome) linkage groups were numbered based on colinearity to *A*. *duranensis* (A-genome) linkage groups except with the suffix “B”. The colinear subgroups in *A*. *batizocoi* were named by identical numbers with numerical suffixes, while an un-separated linkage group was identified by using linkage group numbers from the fused groups.

## Competing interests

The authors declare that they have no competing interests.

## Authors’ contributions

YG led the experiments and drafted the manuscript. SK, ST, JEB, AFH, NK, EDN, DZ, CAT, and HTS participated in the experiments. POA and SJK designed experiments, coordinated the study, POA finalized the manuscript. All authors read and approved the final manuscript.

## Supplementary Material

Additional file 1 Summary of EST database for SSR discovery. Click here for file

Additional file 2 List of peanut EST-SSRs. Click here for file

Additional file 3 Overview of EST-SSR amplification. Click here for file

Additional file 4 Summary of marker polymorphism among peanut genotypes. Click here for file

Additional file 5 Effect of SSR length on frequency of polymorphism among tetraploid and diploid genotypes. Click here for file

Additional file 6 Arachis germplasm screened for genomic SSR markers developed from GSS sequences. Click here for file

Additional file 7 Peanut genomic SSR primer pair sequences used in this study. Click here for file

Additional file 8 The distribution of distorted loci along linkage groups for the B- genome map. Click here for file
