# Supplementary material for: Comparative mapping in intraspecific populations uncovers a high degree of macrosynteny between A- and B-genome diploid species of peanut
Source: BMC Genomics. 2012 Nov 10;13:608. doi: 10.1186/1471-2164-13-608 (PMC3532320; doi:10.1186/1471-2164-13-608)
Supplement: Additional file 1 — Summary of EST database for SSR discovery. [file 1471-2164-13-608-S1.doc]

**Additional file 1.** Summary of EST database for SSR discovery. Tetraploid peanut ESTs (GenBank Acc. No. CD037499-CD038843, ES702769-ES768453, GO256999-GO269325, GO322902- GO343529 and short-read Sequence Read Archive accessions SRX020012, SRX019979, SRX019972 , SRX019971) were assembled into 101,132 unigenes (37,916 contigs (GenBank Acc. No. EZ720985-EZ758900) and 63,216 singletons).

| Library name | Sequence methods | Library type | tissue | No of sequences | contributor | Genotype |
| --- | --- | --- | --- | --- | --- | --- |
| VBL1 | Sanger | EST | Leaves and cotyledons | 12,210 | NCSU | Tifrunner |
| TFL | Sanger | EST | Leaves | 6,330 | USDA-Tifton | Tifrunner |
| TFR5 | Sanger | EST | R5 developing seeds | 6,319 | USDA-Tifton | Tifrunner |
| TFR6 | Sanger | EST | R6 developing seeds | 4,390 | USDA-Tifton | Tifrunner |
| TFR7 | Sanger | EST | R7 developing seeds | 2,100 | USDA-Tifton | Tifrunner |
| VBL6 | Sanger | EST | Developing embryos | 20,148 | NCSU | Tifrunner |
| C20L | Sanger | EST | Leaves | 8,446 | USDA-Tifton | GT-C20 |
| C20R5 | Sanger | EST | R5 developing seeds | 4,785 | USDA-Tifton | GT-C20 |
| C20R6 | Sanger | EST | R6 developing seeds | 2,019 | USDA-Tifton | GT-C20 |
| C20R7 | Sanger | EST | R7 developing seeds | 2,039 | USDA-Tifton | GT-C20 |
| CVPL | Sanger | EST | Leaves | 483 | USDA-Tifton | A13 |
| NMPL | Sanger | EST | Leaves | 249 | USDA-Tifton | A13 |
| ISBL | Sanger | EST | Leaves | 1,061 | USDA-Tifton | A13 |
| AH | Sanger | EST | Leaves | 192 | USDA-Tifton | A13 |
| NMVA | 454 FLX | EST 454 FLX | Developing seeds | 82,782 | UGA | New Mexico Valencia |
| NMVA | 454 FLX | EST 454 FLX | Developing seeds | 67,560 | UGA | New Mexico Valencia |
| NC12C | 454 FLX | EST 454 FLX | Developing seeds | 72,436 | UGA | NC12C |
| NC12C | 454 FLX | EST 454 FLX | Developing seeds | 48,181 | UGA | NC12C |
