# Supplementary material for: Comparative mapping in intraspecific populations uncovers a high degree of macrosynteny between A- and B-genome diploid species of peanut
Source: BMC Genomics. 2012 Nov 10;13:608. doi: 10.1186/1471-2164-13-608 (PMC3532320; doi:10.1186/1471-2164-13-608)
Supplement: Additional file 3 — Overview of EST-SSR amplification. [file 1471-2164-13-608-S3.docx]

**Additional file 3.** Overview of EST-SSR amplification.

| Amplification | No. of markers | Percentage |
| --- | --- | --- |
| No amplification | 273 | 12.8% |
| Poor amplification* | 55 | 2.6% |
| Yield scorable amplicons < 1,200 bp | 1,768 | 83.0% |
| Amplicons > 1,200 bp | 42 | 2.0% |
| Total | 2138 |  |

*SSR markers with weak amplification and/or multiple bands

Data were generated based on screening of eight genotypes: Tifrunner (PI 644011), GTC20, SunOleic 97R, NC94022, DUR25 (PI 475887 ), DUR2 (Grif 15036), BAT9 (PI 468327), BAT12 (PI 298639).
