# Supplementary material for: Comparative mapping in intraspecific populations uncovers a high degree of macrosynteny between A- and B-genome diploid species of peanut
Source: BMC Genomics. 2012 Nov 10;13:608. doi: 10.1186/1471-2164-13-608 (PMC3532320; doi:10.1186/1471-2164-13-608)
Supplement: Additional file 4 — Summary of marker polymorphism among peanut genotypes. [file 1471-2164-13-608-S4.docx]

**Additional file 4.** Summary of marker polymorphism among peanut genotypes with EST-SSR markers

| Genotype combinations | No. of polymorphic markers | Percent of polymorphism (total markers) | Percent of polymorphism (scorable markers) |
| --- | --- | --- | --- |
| Tifrunner and GTC20 | 157 | 7.4% | 8.9% |
| SunOleic 95R and NC94022 | 126 | 5.9% | 7.1% |
| Four tetraploid lines (any combination) | 240 | 11.2% | 13.6% |
| 30081 and 9484 | 455 | 21.3% | 25.7% |
| PI 475887 and Grif 15036 | 896 | 41.9% | 50.7% |
| Four diploid lines (any combination) | 1450 | 67.8% | 82.0% |
| Four diploid and four tetraploid lines | 1596 | 74.7% | 90.3% |
