# Supplementary material for: Comparative mapping in intraspecific populations uncovers a high degree of macrosynteny between A- and B-genome diploid species of peanut
Source: BMC Genomics. 2012 Nov 10;13:608. doi: 10.1186/1471-2164-13-608 (PMC3532320; doi:10.1186/1471-2164-13-608)
Supplement: Additional file 5 — Effect of SSR length on frequency of polymorphism among tetraploid and diploid genotypes. [file 1471-2164-13-608-S5.pdf]

A.

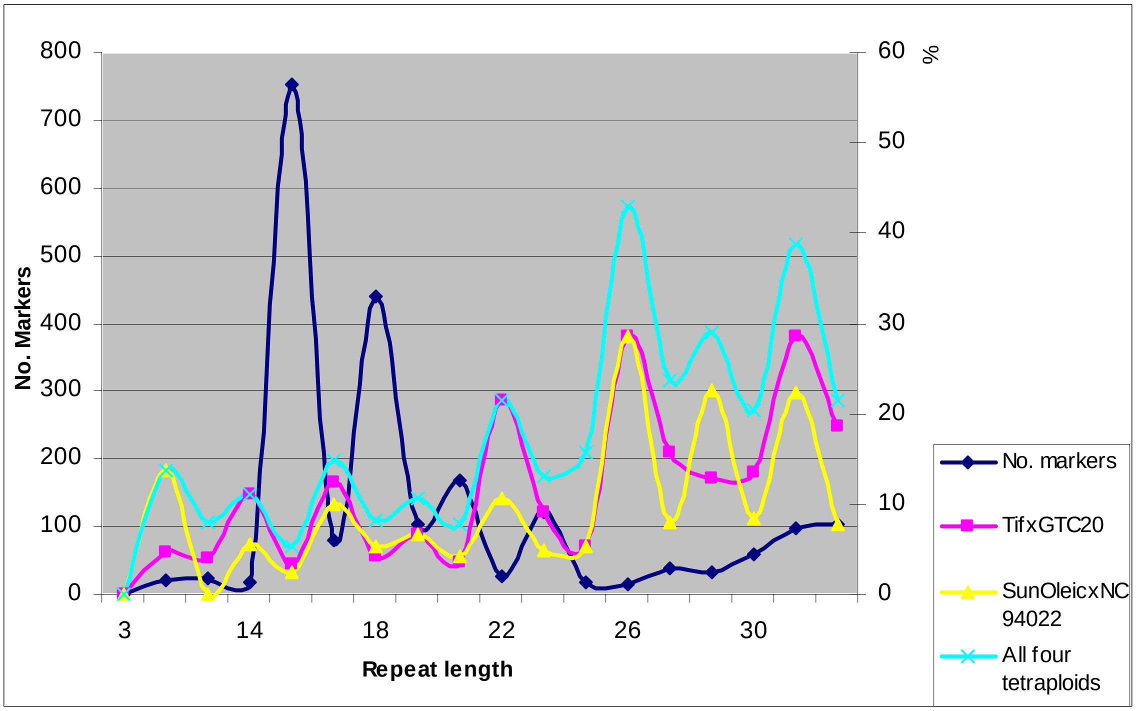

B.

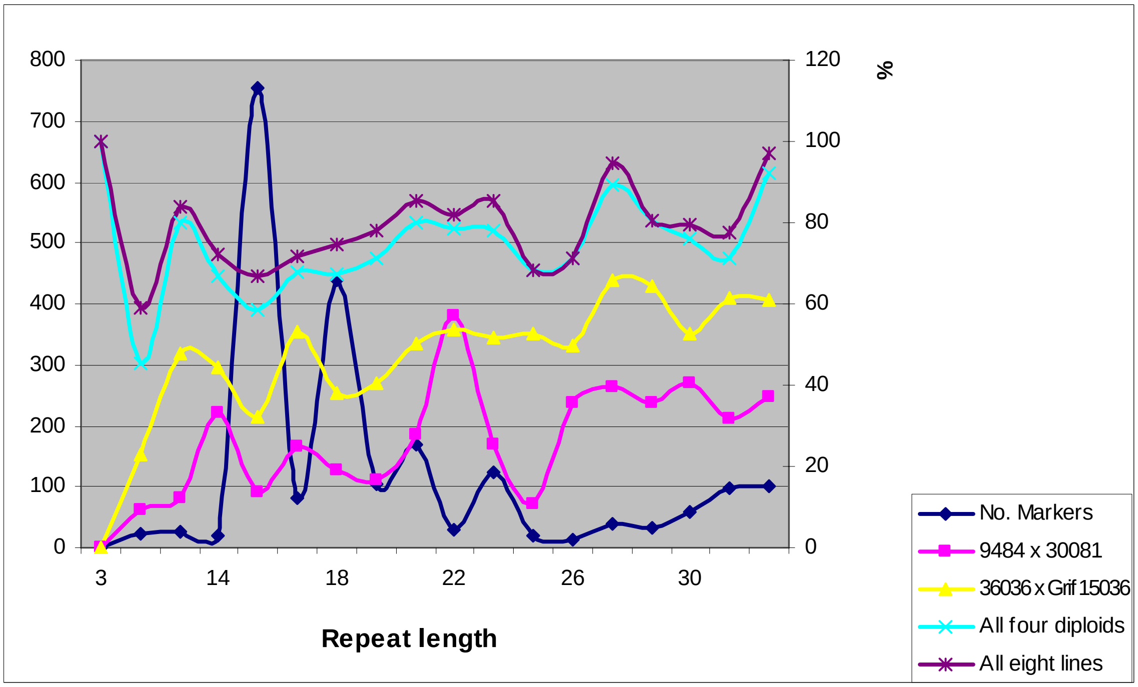

Additional file 5. Effect of SSR length on frequency of polymorphism among tetraploid and diploid genotypes.
