# Supplementary material for: Comparative mapping in intraspecific populations uncovers a high degree of macrosynteny between A- and B-genome diploid species of peanut
Source: BMC Genomics. 2012 Nov 10;13:608. doi: 10.1186/1471-2164-13-608 (PMC3532320; doi:10.1186/1471-2164-13-608)
Supplement: Additional file 6 — Arachis germplasm screened for genomic SSR markers developed from GSS sequences. [file 1471-2164-13-608-S6.doc]

**Additional file 6**. *Arachis* germplasm screened for genomic SSR markers developed from GSS sequences.

| **Scientific Name** | **Accession ID or**  **Cultivar Name** | **PI Number** |
| --- | --- | --- |
| *A. duranensis* Krapov. and W. C. Greg. | 36036 | PI 475887 |
| *A. duranensis* Krapov. and W. C. Greg. | 38901 | PI 497483 |
| *A. duranensis* Krapov. and W. C. Greg. | 30078 | PI 468324 |
| *A. batizocoi* Krapov. and W. C. Greg. | 30080 | PI 468326 |
| *A. hypogaea* var. *aequatoriana* Krapov. and W. C. Greg. |  | PI 497630 |
| *A. hypogaea* var. *fastigiata* (Waldron) Krapov. & W. C. Greg. |  | PI 497471 |
| *A. hypogaea* var. *hirsuta* J. Kohler |  | PI 576613 |
| *A. hypogaea* var. *hypogaea* | Tifrunner | PI 644011 |
| *A. hypogaea* var. *peruviana* Krapov. and W. C. Greg. |  | PI 502045 |
| *A. hypogaea* var. *fastigiata (Waldron)* Krapov. & W. C. Greg. | GT-C20 | - |
| *A. hypogaea* L. | GT-C9 | - |
| *A. hypogaea* L. | A100 | - |
