# Supplementary figures and images for: Comparative mapping in intraspecific populations uncovers a high degree of macrosynteny between A- and B-genome diploid species of peanut
Source: BMC Genomics. 2012 Nov 10;13:608. doi: 10.1186/1471-2164-13-608 (PMC3532320; doi:10.1186/1471-2164-13-608)

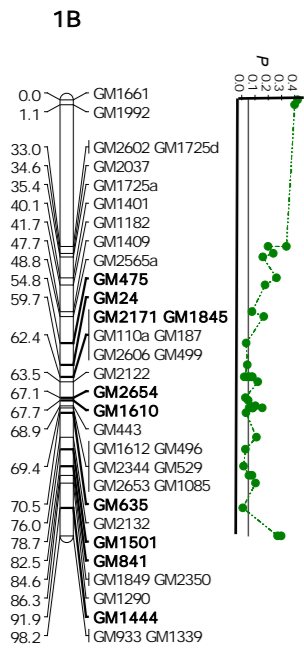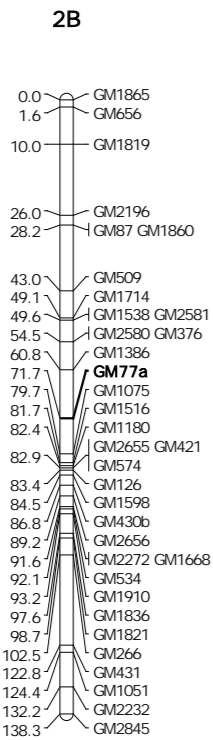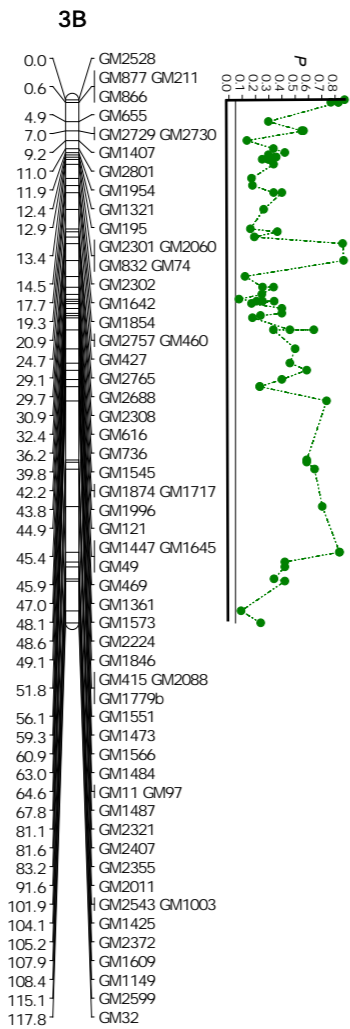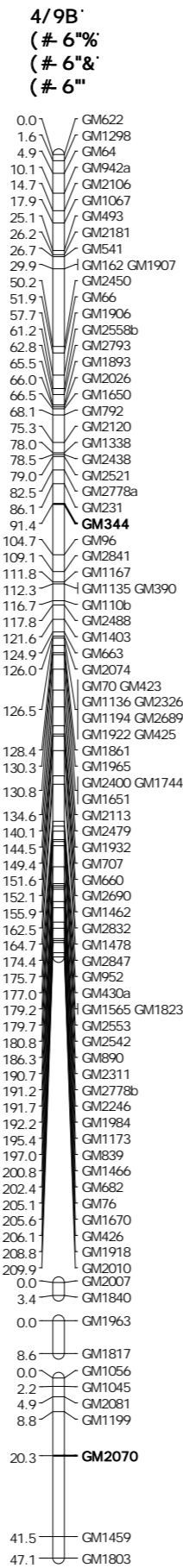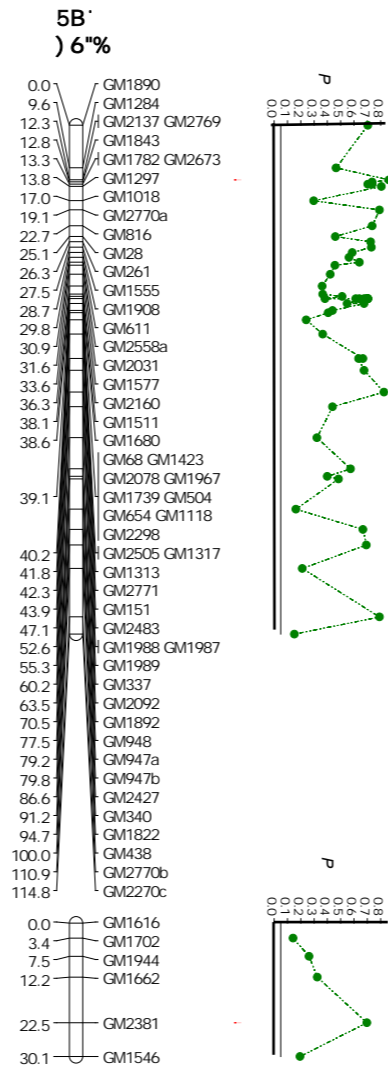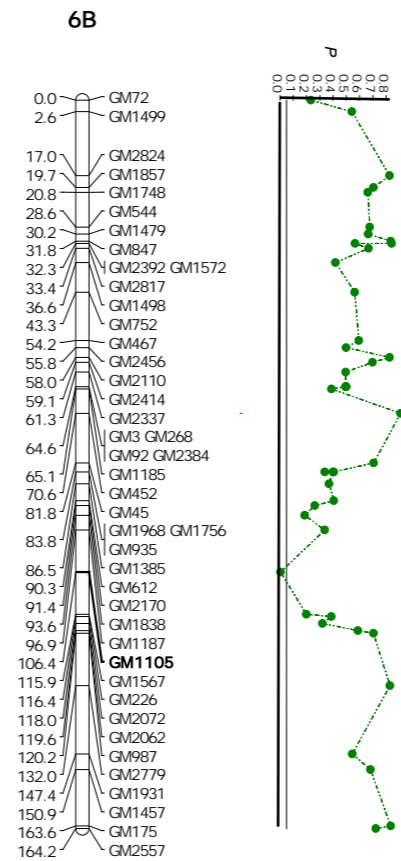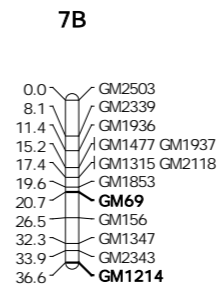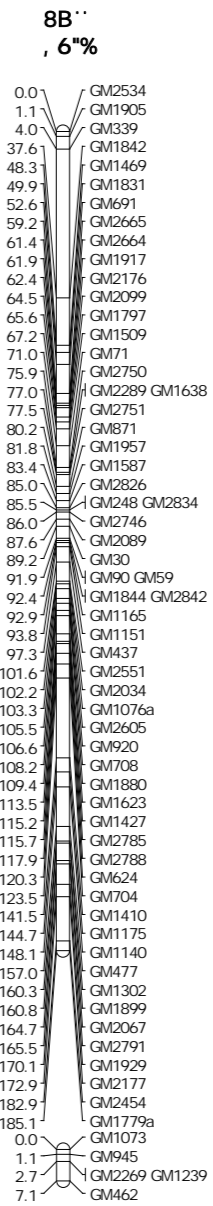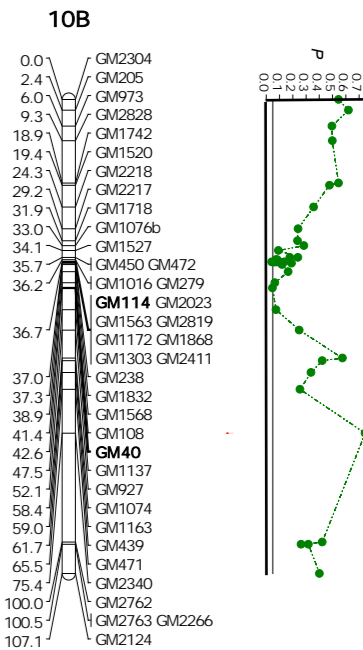

Supplement: Additional file 8 — The distribution of distorted loci along linkage groups for the B- genome map. [file 1471-2164-13-608-S8.pdf]
